# Improved Estimation of Left Ventricular Volume from Electric Field Modeling

**DOI:** 10.2478/joeb-2021-0015

**Published:** 2021-12-27

**Authors:** Leonie Korn, Stephan Dahlmanns, Steffen Leonhardt, Marian Walter

**Affiliations:** 1 Medical Information Technology, RWTH Aachen University, Aachen, Germany

**Keywords:** Left ventricular volume, Electric field distribution, LVAD

## Abstract

Volume measurement is beneficial in left ventricular assist device (LVAD) therapy to quantify patient demand. In principle, an LVAD could provide a platform that allows bioimpedance measurements inside the ventricle without requiring additional implants. Conductance measured by the LVAD can then be used to estimate the ventricular radius, which can be applied to calculate ventricular volume. However, established methods that estimate radius from conductance require elaborate individual calibration or show low accuracy.

This study presents two analytical calculation methods to estimate left ventricular radius from conductance using electric field theory. These methods build on the established method of Wei, now considering the dielectric properties of muscle and background tissue, the refraction of the electric field at the blood-muscle boundary, and the changes of the electric field caused by the measurements.

The methods are validated in five glass containers of different radius. Additional bioimpedance measurements are performed in in-vitro models that replicate the left ventricle’s shape and conductive properties. The proposed analytical calculation methods estimate the radii of the containers and the in-vitro models with higher accuracy and precision than Wei’s method. The lead method performs excellently in glass cylinders over a wide range of radii (bias: 1.66%–2.48%, limits of agreement < 16.33%) without calibration to specific geometries.

## Introduction

The use of left ventricular assist devices (LVADs) in the treatment of advanced heart failure or cardiogenic shock has proven to improve patient outcomes significantly [1, 2, 3]. The advanced monitoring of pressures and flows in the circulation would allow to adapt LVAD therapy to individual needs and reduce adverse events [4, 5]. In the past, various control strategies of an LVAD that adapt to the loading conditions of the heart measured by different sensor modalities were studied by several groups [6, 7, 8, 9, 10, 11] without being clinical routine. In general, reliable monitoring of hemodynamics has been shown to reduce rehospitalization [12]. In particular, left ventricular volume (LVV) is one potential input variable for physiological VAD control [13, 14]. Bioimpedance measurements have been identified as a potential tool for the assessment of LVV [15]. As shown previously by [14, 16], an implanted LVAD can even provide a suitable platform for sensor integration.

Techniques for intracardiac bioimpedance measurement have been developed over the last 40 years. The invasive nature of the LVAD can be utilized to place the measurement setup, including the electrodes directly into the heart, without the need for additional surgical access to the patient. The established methods of Wei et al. [17], and Baan et al. [18, 19] sought to derive an analytical estimate of the left ventricular radius from the electric field distribution spanned inside the ventricle. LVV can then be calculated from this radius using ventricular volume models. The methods of Wei and Baan are regarded as the gold standard in intracardiac bioimpedance measurement, in research, and commercially available pV-loop catheters but are not clinical routine.

Both methods are based on a multitude of simplifying assumptions regarding the electric field distribution inside the heart caused by bioimpedance measurements. Baan et al. [18] assume a linear conductance-volume relationship. This results in a false assumption of the dependency between impedance and cylindrical volume segments. Furthermore, the methods by Baan and Wei neglect the boundary to the conductive myocardium and background tissues. Lastly, only the electric field distribution of the excitation field is modeled, and the measurement field is omitted, which contradicts the reciprocity condition [20]. As a result, both methods overestimate LVV [18, 21, 22]. In addition, Kass et al. [23] found that changes in conductance are very small over one heart cycle in patients with dilated hearts and reduced ejection fraction, which is not represented by these methods. The experimental results from Wu et al. [22] support this conclusion.

Wei et al. also provide an empirical solution to determine LVV. However, this approach necessitates, in addition to a given blood conductivity, patient-specific geometric calibration using echocardiographic volumetry. Further, parallel muscle conductance significantly influences absolute LVV measurements [21]. As the refraction of the electric field at the muscle boundary is not sufficiently considered by Baan and Wei, its proportion needs to be elaborately determined and then subtracted from the measurement signal [24, 25, 26, 19].

This study aims to establish new relationships between the geometric properties of the left ventricle and the distribution of the electric field generated by intracardiac bioimpedance measurements, simplifying and improving the assessment of LVV.

## Analytical calculation methods

This section presents the analytical calculation methods (ACM) developed to improve absolute LVV estimation. In this study, we focus on tetrapolar measurement configurations. Larson et al. [27] found that tetrapolar configurations show better sensitivity distribution in the blood cavity compared to the commonly used multipolar configurations.

Wei et al. [17] postulated a nonlinear conductance-volume relationship designed and validated for infinite and homogeneous blood media.

**Figure 1:**
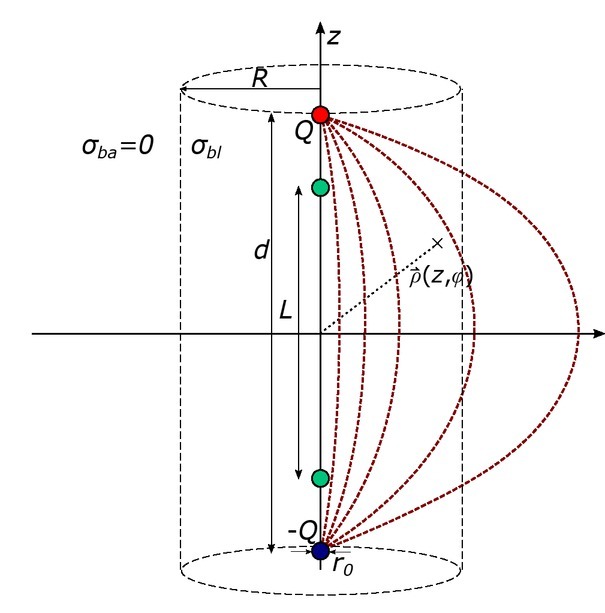
Electric field distribution of to spheres with opposite charges *Q* (red) and -*Q* (blue). The measurement electrodes (green) are distanced by *L*. Background tissue (σ_ba_ = 0) is assumed.


The analytical solution of this relationship was derived by modeling the electric field of two point charges, as depicted in Fig. 1, From electric field theory, the electric field distribution ~E_sup_ of a superposition of two point charges in cylindrical coordinates (, , z) is given by

(1) $$\begin{array}{l} {\vec{E}}_{\text{sup }}=\\ \frac{Q}{4\pi \epsilon }\cdot \left(\frac{\left(z-\frac{d}{2}\right)\cdot {\vec{e}}_{z}+\rho \cdot {\vec{e}}_{\rho }}{{\sqrt{{\left(z-\frac{d}{2}\right)}^{2}+\rho^{2}}}^{3}}-\frac{\left(z+\frac{d}{2}\right)\cdot {\vec{e}}_{z}+\rho \cdot {\vec{e}}_{\rho }}{\sqrt{{\left(z+\frac{d}{2}\right)}^{2}+\rho^{2}}}\right), \end{array}$$

assuming that the two charges ±Qare located at $z=\frac{d}{2}$and $Z=-\frac{d}{2}.$The distance d between the charges represents the distance between the injection electrodes in a tetrapolar setup. The current I_Wei_ that is induced by this dipole arrangement is determined integrating the current density $\vec{J}=\sigma_{\text{bl}}\cdot {\vec{E}}_{\text{sup }}$in the z-plane at z = 0 [28],


(2) $$\begin{array}{l} {/}_{\text{Wei}}=\int_{\phi =0}^{2\pi }\int_{\rho =r_{0}}^{R}\sigma_{\text{bl}}\cdot {\vec{E}}_{\text{sup },\text{z}}(z=0)\;\text{d}\vec{A}\\ =\frac{Q}{4\pi \epsilon }\cdot 4\pi \sigma_{\text{bl}}\cdot d\cdot \left(\frac{1}{\sqrt{d^{2}+4R^{2}}}-\frac{1}{\sqrt{d^{2}+4r_{0}^{2}}}\right), \end{array}$$

with dA = dd in cylindrical coordinates. R marks the radius of the blood cavity, r_0_ describes the diameter of the injection electrodes, and σ_bl_ the conductivity of blood.

The voltage drop V_Wei_ is described by the integration of E~_sup_ between the two measurement electrodes along the z-axis [28]. Assuming a distance L between the measurement electrodes, it follows,


(3) $$V_{\text{Wei}}=-\int_{-\frac{L}{2}}^{\frac{L}{2}}E_{\text{sup }}dz=-4\cdot \frac{Q}{4\pi \epsilon }\cdot \frac{2L}{d^{2}-L^{2}}.$$

Finally, the conductance G_Wei_ of the left ventricle is calculated using Ohm’s law,


(4) $$\begin{array}{rl} & G_{\text{Wei }}=\frac{I_{\text{Wei }}}{V_{\text{Wei }}}\\ & \;\;\;\;\;\;\;\;\;\;\;\;=\frac{\pi \sigma_{\text{bl}}\left(d^{2}-L^{2}\right)}{4L}\cdot \left(\frac{1}{\sqrt{d^{2}+4r_{0}^{2}}}-\frac{1}{\sqrt{d^{2}+4R^{2}}}\right), \end{array}$$

which is similar to the result from [17]. Applying Eq. 4, R can be derived based on the known geometric properties of the measurements setup and the measured G_Wei_, Wei et al. then applied a cylindrical volume model (LV V _Wei_ = R^2^ L) to estimate LVV [17].

Building on this ACM to estimate R from G, we repeal some of its simplifications and propose two new ACMs for improved estimation of the ventricular radius from bioimpedance measurements.

### Plate ACM

The electric field refracts at the boundary of two materials (1 and 2) with conductivities σ1 and σ2, respectively. For the normal and tangential components of the current density fields J1 and J2 applies [28]


(5) $$J_{1n}=J_{2n}\;\;\;\text{and}\;\;\;\;J_{1t}\cdot \sigma_{2}=J_{2t}\cdot \sigma_{1}.$$

In order to consider these dependencies in an analytical description of the electric field distribution, the incident angles at each point of the boundary have to be known. However, the initial fields E1 and E2 are unknown and thus an analytical solution is not derivable.

Assuming a high conductivity of material 1 and a low conductivity of material 2 $\left(\sigma_{1}>\sigma_{2}\right),$the current density is refracted in tangential direction for material 1 according to Eq. 5, As a result, the field is distorted and compressed into the high conducting material. Based on this observation, we assume that in intracardiac bioimpedance measurements the field is compressed into the blood-filled cavity, as blood is more conductive than muscle tissue (σ_bl_ = 0.7 S/m > σ_m_ = 0.17 S/m at 20 kHz, see [29]).

**Figure 2:**
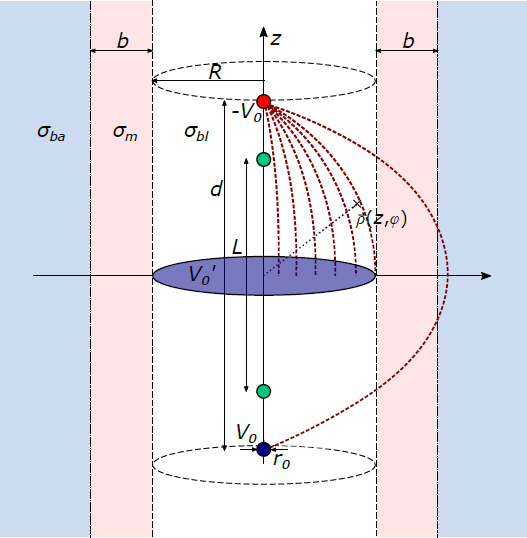
Electric field distribution by applying the voltages -V_0_ (red) on a sphere and $V_{0}^{\prime }=+V_{0}$on a spare charge plate (blue). The measurement electrodes (green) are distanced by *L*. Muscle tissue (σ_m_) with a thickness of b and background tissue (σ_ba_) are modeled.

Thus, we propose to model the refraction of the electric field by superposing the electric field of one charged sphere $Q\;\text{at}\;Z=\frac{d}{2}$with the electric field of one charged plate $-Q^{\prime }\;\text{at}\;z=0\left({\vec{E}}_{\text{pla }}\right).$This arrangement is depicted in Fig. 2, The size of the plate is equal to the cross-sectional area of the blood cavity with radius R, Due to its equally distributed charge and its bounded size, the electric field is compressed into the blood filled volume.

The plate carries the same charge as the sphere, which has a voltage of V0 and the radius r0,


(6) $$\frac{-Q^{\prime }}{4\pi \epsilon }\overset{!}{=}\;V_{0}\cdot r_{0}.$$

Here, ⌈Q^0^ is equally distributed on the plate. E~_pla_ is then defined by [28]


(7) $${\vec{E}}_{\text{pla}}=V_{0}\cdot r_{0}\cdot \frac{2}{R^{2}}\left(1-\frac{z}{\sqrt{z^{2}+R^{2}}}\right){\vec{e}}_{z},$$

It yields the complete field E~_sup,pla_ by a summation of Eq. 7 with the electric field of a sphere at $Z=\frac{d}{2},$


(8) $$\begin{array}{l} {\vec{E}}_{\text{sup,pla }}=-V_{0}r_{0}.\\ \;\;\;\left(\frac{\left(z-\frac{d}{2}\right)\cdot {\vec{e}}_{z}\left(\rho -r_{0}\right){\vec{e}}_{\rho }}{{\sqrt{{\left(z-\frac{d}{2}\right)}^{2}+{\left(\rho -r_{0}\right)}^{2}}}^{2}}-\frac{2}{R^{2}}\left(1-\frac{z}{\sqrt{z^{2}+R^{2}}}\right){\vec{e}}_{z}\right) \end{array}$$

The current I_sup,pla_ excited by this field is the integration of the current density ${\vec{J}}_{\text{sup,pla }}=\sigma_{\text{bl }}\cdot {\vec{E}}_{\text{sup,pla }}$in the z-plane


(9) $$\begin{array}{l} \text{(}z\;\text{=}\;\text{0) analogous to Eq. 2,}\\ I_{\text{sup,pla }}=\\ \;\;-8r_{0}V_{0}\pi \sigma_{\text{bl}}\left(\frac{4r_{0}^{2}-4r_{0}R+d^{2}}{d\sqrt{4r_{0}^{2}-8r_{0}R+4R^{2}+d^{2}}}-2+\frac{r_{0}^{2}}{R^{2}}\right) \end{array}$$

The voltage drop V_sup,pla_ at the location of the measurement electrodes is derived by integrating the electric field along the z-axis considering the symmetry of the setup to the z = 0 plane,


(10) $$\begin{array}{l} V_{\text{sup, pla }}=-2\cdot \int_{0}^{\frac{L}{2}}E_{\text{sup,pla }}\text{dz}=-8r_{0}V_{0}.\\ \;\;\;(\frac{2}{\left(L-d\right)}-\frac{L}{R^{2}}+\frac{2}{R^{2}}\sqrt{{\left(R-r_{0}\right)}^{2}+{\left(\frac{L}{2}\right)}^{2}}\\ \;\;\;+\frac{2}{d}-\frac{2}{R^{2}}\cdot \left(R-r_{0}\right)). \end{array}$$

This yields the following ACM of the conductance G_sup,pla_ of the substitute plate configuration,


(11) $$\begin{array}{l} G_{\text{sup,pla }}=\pi \sigma_{\text{bl}}\\ \frac{\frac{4r_{0}^{2}-4r_{0}R+d^{2}}{d\sqrt{4r_{0}^{2}-8r_{0}R+4R^{2}+d^{2}}}-2+\frac{r_{0}^{2}}{R^{2}}}{\frac{2}{\left(L-d\right)}-\frac{L}{R^{2}}+\frac{2}{d}-\frac{2}{R^{2}}\cdot \left(R-r_{0}\right)+\frac{2}{R^{2}}\sqrt{{\left(R-r_{0}\right)}^{2}+{\left(\frac{L}{2}\right)}^{2}}} \end{array}$$

G_sup,pla_ is a function of parameters of the measurement setup (r_0_, d, L), the geometry of the ventricle (R), and

the blood conductivity (σ_bl_). With an increased distance of the blood-muscle boundary R to the z-axis, the electric field simplifies to the field of two spherical charges as proposed by Wei et al. [17]. This effect is not considered in the plate ACM (cf. Eq. 11), as Q^'^ is equally distributed on the plate. Consequently, the plate configuration should only be applied for small R (R < L). Therefore, the Wei ACM (cf. Eq. 4) is adapted to consider the conductance of muscle and background tissue. This was achieved by extending its integral limits and using the conductivity of muscle/background to calculate the resulting electrical current in Eq. 2, The plate ACM is then used to estimate the conductance of the blood cavity G_sup,pla_, Assuming that G_sup,pla_ can be modeled parallel to the conductance of the muscle G_m_ and background G_ba_, the complete compartment G_plate_ is described by their summation,


(12) $$G_{\text{plate }}=G_{\text{sup, pla }}+G_{\text{m}}+G_{\text{ba }}.$$

### Lead ACM

The second ACM to describe the relationship between the conductance measured and the radius of the ventricle is based on lead field theory. The concept of lead field theory was introduced by Helmholtz et al. [20]. Its fundamental idea is the reciprocity of the measurement and injection field: in tetrapolar measurement configurations, it is irrelevant which pair of electrodes is used for current injection or voltage measurement. The impedance measured is identical for both setups. As a result, the macroscopic impedance was defined in the lead field theory of Malmivuo and Plonsey [30]. Accordingly, the impedance of a tetrapolar arrangement is calculated by the volume integral of the product of the normalized current densities J_I_ and J_M_ of the injection and the measurement field.

Within the ventricle, we model the electric field distributions resulting from either an injection at the inner electrodes, or an injection at the outer electrodes, using the electric field distribution E_sup_ of two spheres (cf. Eq. 1), interchanging the values of d and L, From ~ JI;M = σ ~E, the injection current density J_I_ and the measurement current density J_M_ are then derived.

**Figure 3:**
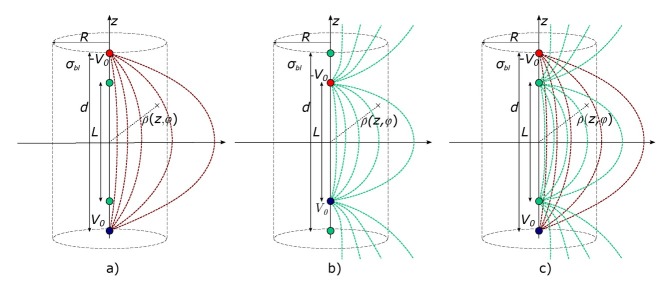
Left: electric field distribution of the injection at the outer electrodes with distance *d*. Middle: electric field distribution of the injection at the inner electrodes with distance *L*. Right: electric field distribution considering both the injection and the measurement field according to lead field theory.

Figure 3 c) schematically shows the superposition of the injection (red) and the measurement field (green). Unfortunately, it is not possible to solve the volume integral over the product of J_I_ and J_M_ analytically. Consequently, we propose a simplified solution to account for the influence of the measurement field.

From reciprocity follows that the conductance measured is independent of interchanges between the injection and measurement electrodes. Hence,


(13) $$G_{\text{d}}\overset{!}{=}G_{\text{L}}$$

needs to be valid. The indices of G indicate the distance between the electrodes used for injection (outside: d, inside: L), whereas the other electrode pair is used for voltage measurement. Considering the configurations shown in Fig. 3 a) and b), the differences in potential V_d_ and V_L_ of both injection locations modeled by two spherical charges are


(14) $$\begin{array}{l} V_{d}=-\int_{-\frac{L}{2}}^{\frac{L}{2}}E_{\text{sph},\text{d}}\text{d}z=-4V_{0}r_{0}\cdot \frac{2L}{L^{2}-d^{2}},\\ V_{L}=-\int_{-\frac{d}{2}}^{\frac{d}{2}}E_{\text{sph},\text{L}}\text{d}z=-4V_{0}r_{0}\cdot \frac{2L}{L^{2}-d^{2}}, \end{array}$$

and thus V_d_ = V_L_ = V_all_ in correspondence to lead field theory.

The currents I_d_ and I_L_ excited by both fields,


(15) $$\begin{array}{l} I_{\text{d}}=\int_{\rho =r_{0}}^{R}\int_{\phi =0}^{2\pi }\sigma_{\text{bl}}\cdot E_{\text{sph},\text{d}}\cdot \rho \;\text{d}\rho \;\text{d}\phi \\ \;\;\;\;\;\;=-4\pi \sigma_{bl}r_{0}V_{0}\cdot \left(\frac{4r_{0}\left(r_{0}-R\right)+d^{2}}{d\sqrt{4{\left(r_{0}-R\right)}^{2}+d^{2}}}-1\right), \end{array}$$

(16) $$\begin{array}{l} I_{\text{L}}=\int_{\rho =r_{0}}^{R}\int_{\phi =0}^{2\pi }\sigma_{\text{bl}}\cdot E_{\text{sph},\text{L}}\cdot \rho \;\text{d}\rho \;\text{d}\phi \\ \;\;\;\;\;=-4\pi \sigma_{b1}r_{0}V_{0}\cdot \left(\frac{4r_{0}\left(r_{0}-R\right)+L^{2}}{L\sqrt{4{\left(r_{0}-R\right)}^{2}+L^{2}}}-1\right), \end{array}$$

however, are not equal and the reciprocity condition of Eq. 13 is not satisfied. Consequently, the assumption that the electric field of the tetrapolar configuration is described by two ideal spheres is not valid. As a result, the description of the currents needs to be adjusted. According to Eq. 13 and Eq. 14, a straightforward solution to fulfill the reciprocity theorem is given by


(17) $$-\frac{I_{\text{d}}}{V_{\text{all}}}\cdot x_{\text{d}}+\frac{I_{\text{L}}}{V_{\text{all}}}\cdot x_{\text{L}}=0,$$

with x_d_ and x_L_ being unknown factors that model the influence of the measurement field on the injection field. This simple solution implies a multiplicative rather than an additive adaptation of the fields. Hence, if no current is injected (I_d_ = I_L_ = 0), no offset current remains. However, Eq. 17 is under-determined. Therefore, the following limitations for x_d_ and x_L_ are defined:

- x_d_ and x_L_ are unit-less,
- the contribution of both currents I_d_ and I_L_ is balanced, as the current densities in lead field theory also contribute equally [30],

$$•\;\;-X_{\text{d}}=X_{\text{L}}^{-1}.$$

These limitations lead to the unique solutions


(18) $$X_{\text{d}}=\frac{\sqrt{I_{\text{L}}}}{\sqrt{I_{\text{d}}}}\;\;\;\text{ and}\;\;\;\;X_{\text{L}}=\frac{\sqrt{I_{\text{d}}}}{\sqrt{I_{\text{L}}}}.$$

From this directly follows that the modeled current I_lead_ is


(19) $$I_{\text{lead }}=\sqrt{I_{\text{L}}\cdot I_{\text{d}}}$$

This results in the ACM of the conductance G_lead,bl_ of the blood cavity,


(20) $$\begin{array}{l} G_{\text{lead,bl }}=\frac{I_{\text{lead }}}{V_{\text{all }}}=-\pi \sigma_{\text{b}l}.\\ \frac{\sqrt{\left(\frac{4r_{0}\left(r_{0}-R\right)+d^{2}}{d\sqrt{4{\left(r_{0}-R\right)}^{2}+d^{2}}}-1\right)\left(\frac{4r_{0}\left(r_{0}-R\right)+L^{2}}{L\sqrt{4{\left(r_{0}-R\right)}^{2}+L^{2}}}-1\right)}}{\frac{2L}{L^{2}-d^{2}}}. \end{array}$$

The complete conductance G_lead_ further depends on the properties of muscle and background tissue. We assume that the lead calculation is valid for all R, so that the total conductance is calculated by


(21) $$G_{\text{lead }}=G_{\text{lead,bl }}+G_{\text{lead,m }}+G_{\text{lead,ba }}.$$

G_lead,m_ and G_lead,ba_ are obtained by changing the conductivities and the integral limits for current estimation (cf. Eq.16 and 15) according to the muscle and background dimensions.

The relationship between R and G is given for the plate and the lead ACM in Eqs. 11–12 and Eqs. 20–21, respectively, where G is directly depended on R, However, the analytical conversion to R from G is impossible. Hence, the dependence of R from G is described by numerically derived characteristic lookup tables. These tables are based on the model descriptions of the R-dependent conductances, including the geometry of the tetrapolar setup, the conductivity of the blood, and the background conductivity. Dependent on the selected electrode configuration, a specific radius R can be targeted within the ventricle. Hence, from the estimation of R, a volume model as proposed byWyatt et al. [31] can then be applied for the estimation of LVV. Assuming that R is measured in the center of the ventricle, the volume would only be dependent on the height LV_h_ of the ventricle:


(22) $$LVV=\frac{5}{6}\cdot \pi \cdot R^{2}\cdot LV_{h}.$$

## Validation methods

This section introduces the models that are applied to validate the ACMs. One highly controllable setup with glass containers and a setup with two in-vitro silicone phantoms are presented. Tetrapolar bioimpedance measurements are performed with a device described in Korn et al.[32].

### Glass containers

**Figure 4:**
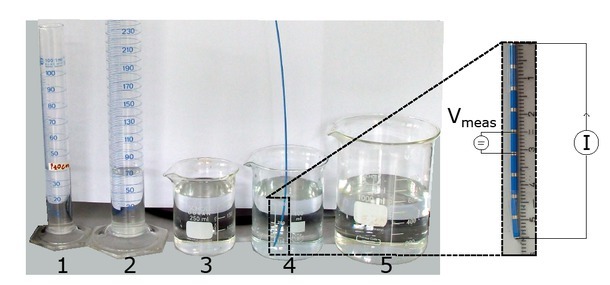
Left: five cylindrical glass containers representing simplistic volumes to test the measurement system and calculation methods. 1: radius 13.3 mm, 2: radius 17.5 mm, 3: radius 32.8 mm, 4: radius 38.6 mm, 5: radius 50.4 mm. Right: close-up on the measurement catheter and a possible cascaded measurement configuration.

Initial testing of the ACMs was performed in cylindrical glass containers. Their cross-sectional areas correspond to a perfect circle and thus fit the assumptions of radius estimation. Five glass containers with radii between 13.3mm and 50.4mm were selected as shown in Fig. 4 to represent a variety of physiological and pathological heart radii [33].

All containers were filled with saline solution to conduct impedance measurements. Two distinct concentrations of saline solutions were prepared to model high conductive and low conductive materials. The solutions’ conductivity was measured with the conductivity meter HI 8733 (HANNA Instruments, Woonsocket, Rhode Island, USA) as σ_1_ = 1.68 S/m and σ_2_ = 0.62 S/m.

### In-vitro phantoms

The glass containers do not accurately replicate ventricle anatomy and omit background material. Thus, the ACMs were tested in in-vitro phantoms to demonstrate proof-of-concept. This reduces animal trials and is in accordance with the 3R strategy [34].

**Figure 5:**
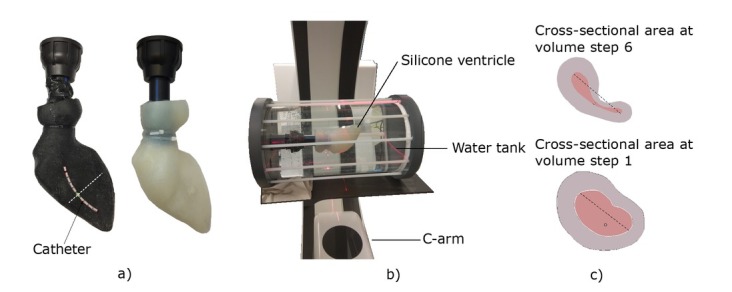
a) Left ventricular in-vitro phantoms made from silicone (left: carbon, right: pure), b) C-arm with manual test bench to measure phantom dimension, c) Cross-sectional areas obtained from CT measurements at the center electrode pair, adapted from [32].

The in-vitro phantoms were cast from silicone (A00, Silikonfabrik.de, Ahrensburg, Germany) and model the anatomy of the left ventricle derived from a left ventricle CT image series from the embodi3D database [35]. The silicone phantoms were either of pure (insulating) silicone or are enriched with carbon additives to replicate the conductive behavior of cardiac tissue [32]. The conductivity of the conductive phantom is approximately σ_m_ = 0.3 S/m, but not exactly determinable due to inhomogeneity in mixing (similar to real muscle tissue). Both phantoms are shown in Fig. 5 a) and were placed inside a tank. A syringe was used to displace water from the tank, which is then inversely displaced inside the phantom.

For each phantom, a displacement of about 10 mL was realized in five steps, which resulted in a cumulative reduction of saline solution in the phantom of approximately 50 mL. The saline solution exhibited a conductivity of σ_bl_ = 0.7–0.8 S/m. The phantom was placed inside a water tank filled with a conductivity of σ_ba_ = 0.2 S/m to replicate background tissue. The volumes of the phantoms were obtained from three-dimensional (3D) computer tomographic (CT) measurements from the silicone phantom as shown in Fig. 5 b). The equivalent circular radius was then estimated of the cross-sectional area between the center electrode pair for each volume step (cf.

Fig. 5 c)). Table 1 gives the volumes, the resulting areas, and the equivalent radius of all six volumes. Information on the position and deformation of the measurement catheter is gained from additional CT scans.

### Ethical approval

The conducted research is not related to either human or animal use.

## Results

All radii estimated by the different ACMs are compared to the reference radius by providing the bias (%) as the mean of the difference between the measured and calculated radii, and the limits of 95% agreement (LOA (%)) as 1.96 of the standard deviations of these differences in relation to the mean radius measured.

### Estimation of glass container radius

**Figure 6:**
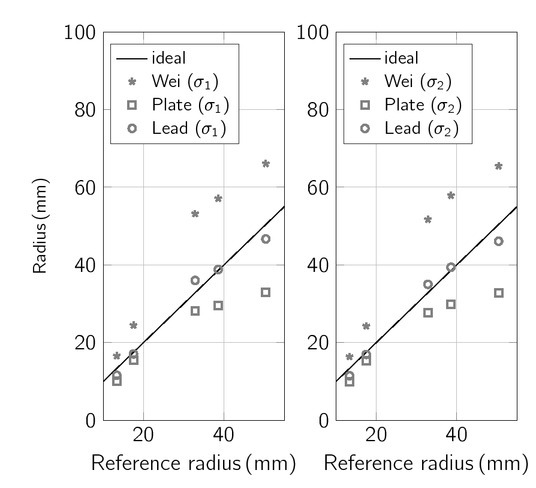
Estimated radii using the classical Wei, the plate, and the lead ACM compared to the known radii of the cylinders (Data). Left: conductivity of saline solution σ1 = 1.69 S/m, right: conductivity of saline solution σ2 = 0.62 S/m.

The conductance measured inside each glass container is applied to the classical Wei ACM [17] (cf. Eq. 4), plate ACM (cf. Eq. 11), and lead ACM (cf. Eq. 20) to estimate the glass container radius. These estimations are depicted in Fig. 6 and compared to the actual radii of the glass cylinders. For all algorithms, the specific conductivity of the saline solution is given.

For small radii (< 20 mm), the lead and the plate ACM estimate radii that are close to the actual radii of the containers within both saline solutions. For all radii, the lead ACM outperforms Wei’s ACM, exhibiting a bias between -1.66% and -2.48% to the reference (bias Wei: 41.35% – 42.43%). The limits of agreement (LOA) are less than 16.33% (LOA Wei: 47.22% – 48.05%). The plate ACM underestimates radius (σ_1_. -23.92% ± 40.01%, σ_2_. -24.39% ± 39.90%). This underestimation is significant for larger radii (container 3–5), but negligible for radii below 20 mm.

The experiment shows that both lead and plate ACM can be used to determine the radius within cylindrical glass containers without further calibration of the algorithms to patient-individual geometric properties of the heart. The lead ACM covers a wide range of radii (13.34mm – 50.4 mm) with an accuracy above 95%, thus including radii of severely dilated ventricles that may appear during

heart failure. Compared to the Wei ACM, the biases are reduced by a factor of ten if the lead ACM is applied. In addition, the plate ACM fits well for radii that are encountered in physiological ranges [33].

### In-vitro phantom measurements

**Figure 7:**
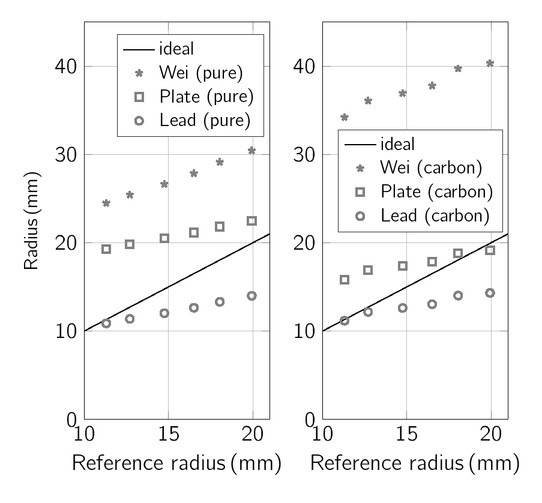
Estimated radii by the ACMs (Wei, Plate, Lead) obtained from the conductances measured inside the in-vitro phantoms compared to reference radii (Data). Left: insulating phantom of pure silicone, right: silicone phantom enriched with carbon.

In this section, the ACMs are applied to estimate the radii of two in-vitro phantoms for six different filling volumes (cf. Tab. 1). Figure 7 shows the estimated radii based on the conductances measured inside the phantoms compared to the center radii of the phantoms determined from 3D CT scans. For all algorithms, we applied measurements of the conductivity of the saline solution (σ_bl_ = 0.7 S/m), either an insulating (σ_m_ = 10^⌈^6 S/m) or conductive myocardium (σ_m_ = 0.3 S/m), and background conductivity (σ_ba_ = 0.2 S/m). These data are applied to the calculation methods to estimate phantom radii.

**Table 1:** Measured volumes inside the silicone ventricle from CT measurements. Cross-sectional areas obtained between the center electrode pair. Estimated equivalent center radius.

Step	Volume (mL)	Area (mm^2^)	Radius (mm)
1	117.90	1248.84	19.94
2	107.12	1022.55	18.04
3	97.96	855.50	16.50
4	87.20	683.15	14.75
5	76.84	506.83	12.70
6	69.46	401.12	11.30

Both the lead and the plate ACM better estimate the radii of both phantoms and all volumes than the Wei ACM, achieving the best results for measurements inside the carbon phantom. This phantom best replicates natural ventricles as it models both the anatomy and the conductivity of the ventricle and background tissue. Here, the radii estimated by the lead ACM deviate by less than 17.1% ± 26.72% (plate: 13.58% ± 26.03%) from the reference values. The application of the Wei ACM leads to a deviation of 141.51% ± 13.75%. For the silicone phantom, the plate and lead ACM biases are increased (plate: 34.14%, lead: -20.41%), the LOAs remain in a range of approximately 26%. The Wei ACM, however, exhibits a smaller difference in the silicone phantom (75.95% ± 12.93%) compared to the carbon phantom, as the silicone phantom better matches the assumptions of Wei et al. [17]. All considered ACM show a dynamic range that is different from the dynamics of the reference radius. The ACMs estimate a lower radius sensitivity on volume than the reference.

## Discussion

The application of the newly proposed ACMs to estimate radius, and hence LVV (cf. 22), from bioimpedance measurements in glass cylinders and in in-vitro ventricle phantoms demonstrates that the plate and the lead ACM outperform the established method of Wei et al. [17]. The measurements inside the glass containers show the possibility of estimating their radii without calibration to patient-individual geometric properties of the heart with an accuracy of more than 95% using the lead ACM. The plate method fits well for radii smaller than 20 mm, but underestimates larger radii. The assumption that the charge _Q_^0^ is uniformly distributed on the plate simplifies this underestimation of larger radii. Consequently, the plate configuration should only be used for smaller radii and is not applicable for highly dilated hearts.

The validation using a carbon-silicone phantom illustrates the importance of considering heart and background conductivity in the ACMs, as the Wei ACM, which assumes insulating muscle, overestimates phantom radius by more than 100%. The plate and lead ACM better estimate radius with biases below 20%.

However, the dynamic changes in radius due to different volumes are not correctly replicated by the ACMs. These discrepancies may be caused by the ACMs or by a non-ideal behavior of the reference radius. CT measurements were not simultaneously conducted with bioimpedance measurements, and catheter position and volume changes may not have been exactly replicated. Therefore, a major challenge of in-vitro validation of the ACMs lies in the precise placement of the electrode catheter. A preliminary study showed that small displacements of the catheter in the radial direction do not significantly affect the results. However, a change of position in the longitudinal direction severely impacts radius estimation. Varying the longitudinal position of the catheter in a phantom with initial volume (step 1) by 1 cm, the impedance measured varies by 8Ωto 38Ω depending on the insertion depth (total insertion depth of 10 cm). Therefore, a fixed longitudinal position should be accounted for, e.g., by screw-in assemblies as used for implantation of pacemaker electrodes or when mounted onto the cannula of the LVAD.

In future work, the proper placement of electrodes in the left ventricle needs to be investigated. Therefore, the carrier for the electrode placement is critical. For example, a catheter-based LVAD has the potential to serve as a platform for the electrodes. If a setup proposed by Cysyk et al. [14] is used, the geometric factors of the electrodes must be adjusted in the proposed ACMS. Another option for electrode placement may be a catheter attached to the cannula that extends from the apex into the left ventricle.

The deformation of the phantom due to volume changes is most likely different from the deformation of a ventricle in vivo. In contrast to the native heart, the walls of a silicone phantom are passively controlled. Therefore, thinner wall sections contribute more to the contraction of the in-vitro model. This unlikely deformation is confirmed by the CT scans of different volume steps (cf. Fig. 5, c)) and might explain the difference of the conductance measured compared to the equivalent circular reference (cf. Fig. 7). Therefore, another approximation of the actual center radius might show fewer differences.

In the future, the deformation of the ventricle should be more realistically represented to validate the accuracy of the proposed ACMs further. Additionally, the integration of electrodes on catheter-based and apex-based LVADS and their influence on the performance of the algorithms needs to be evaluated and tested in in-vivo studies.

## Conclusion

In this study, two new ACMs are proposed that overcome some of the limitations of intracardiac bioimpedance measurements accepted by established methods [17, 18]. The plate and lead ACM account for the background properties of the blood cavity. Additionally, the plate ACM is a workaround to model the refraction of the electric field at the blood-muscle boundary, whereas the lead model considers the contribution of the measurement (lead) field.

By applying these new methods, accuracy in the assessment of radii without calibration to geometric properties is increased from 60% for the established ACM of Wei et al. [17] to more than 95% in glass container experiments. Furthermore, the ventricular radius was estimated with an accuracy above 80% in in-vitro ventricle phantoms. Hence, we suggest using the lead ACM in future measurements of bioimpedance inside the left ventricle, as it covers a wide range of radii. If these estimates are applied to a volume model [31], the proposed ACMs can provide absolute volume assessment necessitating calibration of the longitudinal length only. These improvements in intracardiac bioimpedance measurement may describe a major step forward in LVAD therapy, as clinically established volume measurements from bioimpedance need further calibration to patient-individual stroke volume.

In-vitro silicone models are potent tools for rapidly testing highly invasive measurement techniques before animal experiments are required. They are durable, and the controlled environment allows for a high degree of reproducibility. Future work should focus on accurate reproduction of deformation along the cardiac cycle and measurements in in-vivo trials to further validate the proposed methods.
